# Room light anodic etching of highly doped n-type 4 H-SiC in high-concentration HF electrolytes: Difference between C and Si crystalline faces

**DOI:** 10.1186/1556-276X-7-367

**Published:** 2012-07-03

**Authors:** Gael Gautier, Frederic Cayrel, Marie Capelle, Jérome Billoué, Xi Song, Jean-Francois Michaud

**Affiliations:** 1 GREMAN, UMR CNRS 7347, Université de Tours, 16 rue P. et M. Curie, Tours Cedex 2, 37071, France

**Keywords:** Porous silicon carbide, Electrochemical etching, Si face, C face

## Abstract

In this paper, we study the electrochemical anodization of n-type heavily doped 4 H-SiC wafers in a HF-based electrolyte without any UV light assistance. We present, in particular, the differences observed between the etching of Si and C faces. In the case of the Si face, the resulting material is mesoporous (diameters in the range of 5 to 50 nm) with an increase of the ‘chevron shaped’ pore density with depth. In the case of the C face, a columnar morphology is observed, and the etch rate is twice greater than for the one for the Si face. We've also observed the evolution of the potential for a fixed applied current density. Finally, some wafer defects induced by polishing are clearly revealed at the sample surfaces even for very short etching times.

## Background

Silicon carbide (SiC) has shown huge potential in the field of microelectronic devices. Indeed, this material is characterized by a wide bandgap, high critical electrical field, high electron velocity, and high thermal conductivity. As a consequence, these properties have been employed in various devices where high temperature, high power levels, or high frequencies are required [1]. Among all the polytypes existing for this material, the most common are 6 H, 4 H, 3 C, and 15R. In the case of the hexagonal ones (4 H, 6 H), the strong polar nature of the Si-C bond leads to particular properties of the different crystallographic plans. For instance, the oxidation is clearly anisotropic [2].

The anodic etching of silicon carbide is a well-known technique since the first experiments performed by Shor et al. in 1993 [3]. This technique seems to be a very promising way to etch this material which is very resistant against traditional chemical etching methods. Moreover, porous SiC is known to be an electrical insulating material [4], and its high specific surface allows the fabrication of thick oxide layers by an annealing process [5]. This material has also demonstrated a great potential for epitaxial growth [6].

In this work, we discuss the anodic etching of highly doped (0.03 ohm.cm) n-type 4 H-SiC without any UV lighting contrary to most of the experiments that have been done until now [7,8]. In particular, we explore the morphology differences after etching of Si or C faces. In the last part, we present the polishing defects that can be revealed by the electrochemical etching.

## Methods

### Experimental setup

The main parameters impacting porous semiconductor morphology are the substrate type and doping, the current density or applied voltage, the temperature, the electrolyte composition, and the light intensity and wavelength for n type. In the case of SiC, we must take into account also the influence of the crystalline faces.

**Figure 1:**
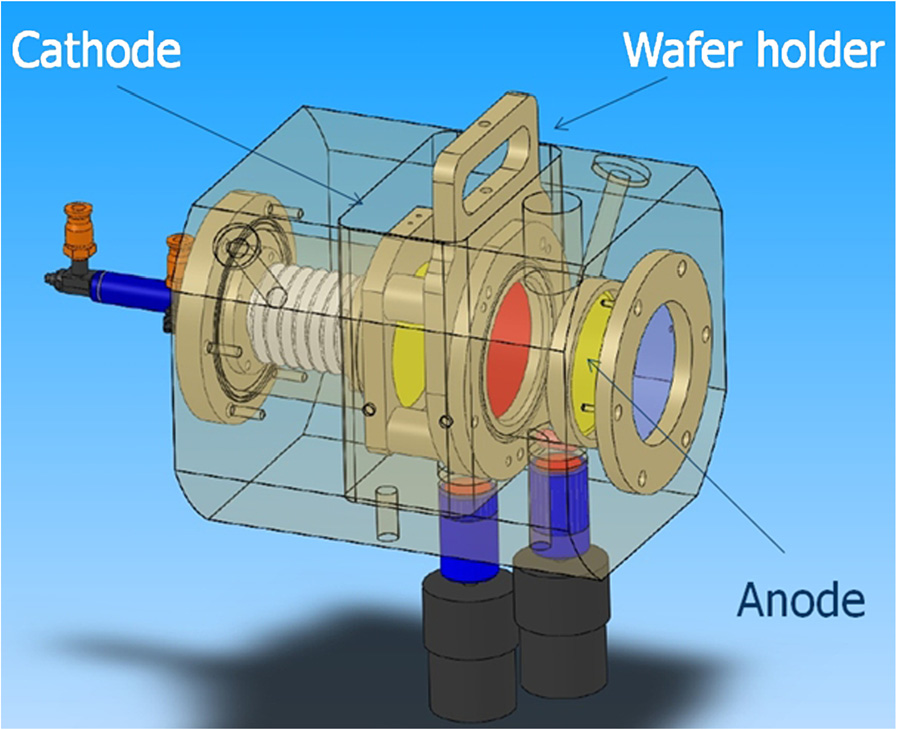
**Double-tank electrochemical cell developed for small samples by AMMT and GREMAN (*Ø***_**aperture**_ **= 1 cm or 1 in.).** The holder is sealed by a pneumatic system. A stirring rod is used to homogenize the electrolyte during the anodization.

The electrochemical etching of our samples was performed in a double-tank electrochemical cell developed by AMMT (Frankethal, Germany) (Figure 1). This equipment was specially designed by our lab to anodize small size samples with diameters ranging from 1 to 15 cm. The backside contact is ensured by the electrolyte. The sample is maintained in a holder which can be illuminated through platinum grid electrodes. However, in our experiments, the anodization was conducted under room light without any additional lighting.

The homogenization of the electrolyte is performed by two stirring rods. This setup helps also to remove the gas bubbles produced during the reaction.

### Anodization conditions

In this study, we used 3-in. 4 H-SiC bulk wafers from Tankeblue® manufacturer (Beijing, China) (4° *off-axis*) in which square samples of 1.5 cm were achieved. The etched area was about 0.79 cm^2^ (*Ø* = 1 cm). The substrate thickness was about 350 μm. The wafers were nitrogen-doped and had a resistivity of 0.037 Ω·cm. The etching was performed under room light without any additional lighting. The duration was fixed at 30 min. The HF concentration is 30%, and the surfactant used was the acetic acid with volume ratios HF(50%):acetic acid:H_2_O of 4.6:2.1:1.5. The anodization was performed in a galvanostatic mode, and the potential between the two platinum electrodes was measured.

The addition of acetic acid as a surfactant in the case of SiC etching is quite unusual. Most of the time, the ethanol is used to increase the electrolyte wettability. However, some authors report the addition of other agents such as Triton X-100 for example [9]. Nevertheless, acetic acid has proven its efficiency in the case of porous silicon etching [10].

The electrochemical etching of SiC is a two-step process. The electrochemical oxidation is described by the Equations 1 and 2 [11]. These reactions involve water and produce silicon oxide if about seven holes are provided to the interface.

(1) $$\text{SiC}+{3\text{H}}_{2}\text{O}+{6\text{h}}^{+}\to {\text{SiO}}_{2}+\text{CO}↑+{6\text{H}}^{+}$$

(2) $$\text{SiC}+{4\text{H}}_{2}\text{O}+{8\text{h}}^{+}\to {\text{SiO}}_{2}+{\text{CO}}_{2}↑+{8\text{H}}^{+}$$

(3) $${\text{SiO}}_{2}+6\text{HF}\to {\text{H}}_{2}{\text{SiF}}_{6}+2{\text{H}}_{2}\text{O}$$

Then, the formed SiO_2_ is chemically dissolved by HF. In our case, the HF concentration can be considered as very high with regard to the values reported in the literature, generally between 5% and 10% [7]. As a consequence, the SiO_2_ dissolution cannot be considered as a limiting factor.

## Results and discussion

In this section, we discuss about the voltage behavior varying the applied potential during the SiC etching. We present also the final morphologies observed with a particular emphasis on the difference between the C and Si faces.

### Evolution of the anodization potential

**Figure 2:**
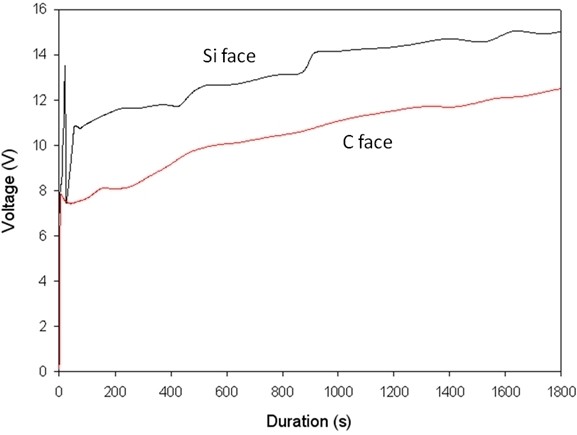
**Measured potential during anodization.** The anodization was done at a fixed current density of 25.5 mA/cm^2^ in the case of the C and Si faces for a 4 H-SiC sample.

**Figure 3:**
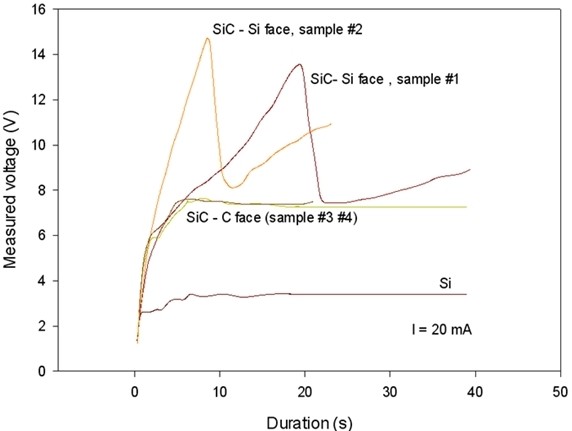
**Measured potential during the initiation of the pores.** The initiation was carried out for a fixed current density of 25.5 mA/cm^2^ in the case of SiC (C and Si faces). Two samples were etched using the same conditions. As a comparison, the curve for a highly doped silicon wafer is also shown. The samples #1, #2 and #3, #4 are identical.

As a first experiment, we applied a constant current density of 25.5 mA/cm^2^ to our system. Figure 2 shows the evolution of the potential during the entire etching process, i.e., for 30 min. A great difference of measured voltage, superior to 2 V, is visible between the two faces. It is well known that the polar character of the Si-C bond has a strong effect on the surface physical properties [12]. In particular, the oxidation rate in a wet or dry ambient can vary significantly [13]. In the case of electrochemical etching, a difference between the two faces was already highlighted by some authors. Indeed, Ke et al. [11] showed, for a constant applied voltage, a larger current density through a C face compared to a Si face. Moreover, a large rise of the potential is clearly visible at the pore initiation (Figure 3) on the Si face. This phenomenon was also observed by Shor et al. [5] in the case of UV-illuminated samples. One can notice that the difference between the two curves obtained for identical conditions is probably due to some discrepancy of the pore initiation process. Moreover, the Si-face potential rise, visible on Figure 3, is not observed on highly doped Si samples, reinforcing the hypothesis of the polarity effect.

The voltage is also increasing in both cases during the experiment. This effect can be correlated with the progressive penetration of the electrolyte in the pores. Indeed, the electrolyte electrical resistivity is about 4 Ω·cm, while the wafer resistivity is two orders of magnitude lower. Moreover, the pore walls are quasi fully depleted. As a consequence, the ohmic resistance increases drastically when the etching front progress into the bulk.

### Observed morphologies

At first, at the opposite of several authors [6], we can observe a variation of the morphology only in depth but in a homogeneous distribution on the etched surface for a current density of 25.5 mA/cm^2^ during 30 min. The average thicknesses of our SiC porous layers were 20 μm and 40 μm, respectively, for the Si and C faces with a dispersion of about 10%. The high etching rates observed for the C face have been also noticed by some authors [7,14]. Nevertheless, in every case, the etching was assisted by illumination. Here, we assume that the holes necessary to produce the SiC oxidation can be generated near the space charge region mainly by tunneling [15,16]. This hypothesis can be confirmed by the low voltages necessary to etch the wafers and the observation of mesoporous morphologies. In addition, if we change the anodization duration, keeping constant the current density, the thickness can be modulated.

**Figure 4:**
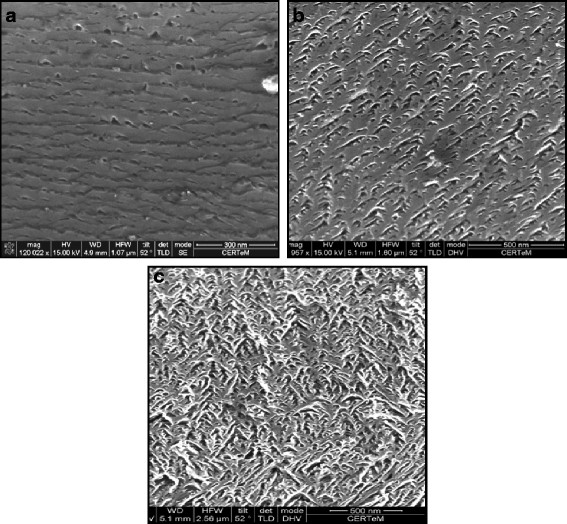
**Evolution of the porous SiC morphology for a constant applied current density of 25.5 mA/cm**^**2**^**.** The observed sample (SEM) was etched from the Si face, and the total layer thickness is about 20 μm. The increase of the porosity and the pore density with the depth is clearly visible. Moreover, (**a**) near the surface, the pores are triangular; (**b**) at a 10-μm depth, they are chevron-shaped; and (**c**) they look like dendrites at 20 μm.

Figure 4 shows the different morphologies observed for the Si face etched with a constant applied current density of 25.5 mA/cm^2^. First, an increase of the porosity and the pore density with the depth is clearly visible. Moreover, near the surface, the pores are triangular (Figure 4a); at a 10-μm depth, they are ‘chevron’-shaped (Figure 4b); and they look like dendrites at 20 μm (Figure 4c).

Some authors have reported pore shrinkage with depth [15], but this effect can only be attributed to the frontside illumination. In fact, in this case, the pores are continuously etched during all the anodization because holes are generated also near the surface. In our case, the holes participating to the reaction are localized near the pore tip. As a consequence, the enlargement (from 20 to 50 nm) and the radical change in the morphology can be attributed to the active species depletion at the pore depth.

**Figure 5:**
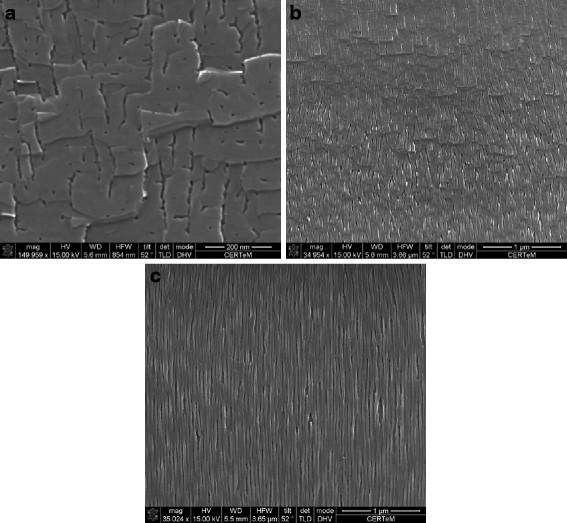
**Evolution of the porous SiC morphology for a constant applied current density of 25.5 mA/cm**^**2**^**.** The cleaved sample was observed (**a**) near the surface and (**b**) at 20-μm and (**c**) 40-μm depths. The observed sample (SEM) was etched from the C face, and the total layer thickness is about 40 μm.

**Figure 6:**
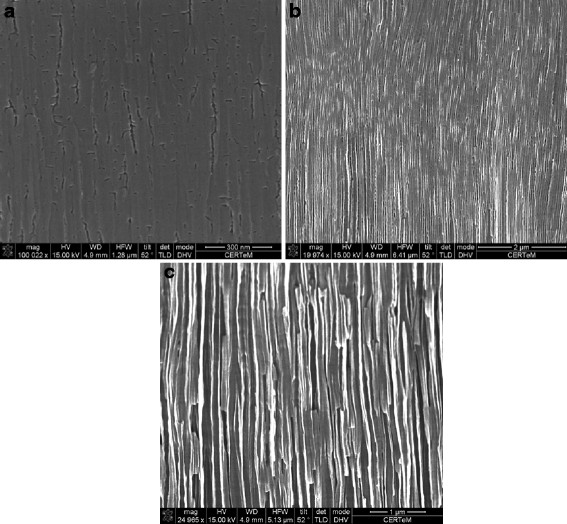
**Evolution of the porous SiC morphology for a constant applied current density of 63.3 mA/cm**^**2**^**.** The cleaved sample was observed (**a**) near the surface and (**b**) at 20-μm and (**c**) 40-μm depths. The observed sample (SEM) was etched from the C face, and the total layer thickness is about 50 μm.

In the case of the C face, this phenomenon also occurs (Figure 5). Nevertheless, for the same conditions, the morphologies are fundamentally different. Near the surface, the pores are clearly branched but well organized following the [0001] direction (Figure 5a). With increasing depth, the pores become larger and the secondary branches disappear (Figure 5b,c). The pore size is about 10 nm near the surface and between 20 and 50 nm at a 40-μm depth. If the current density is increased to 63 mA/cm^2^, the pores are wider with a maximum diameter of about 200 nm at a 40-μm depth (Figure 6).

After a short initiation process and growth of the pores until they reach a steady state, the electrochemical etching is governed by the ratio between the oxidation reaction and the oxide removal rates. The first one is governed by the hole diffusion to the electrolyte/SiC interface. The second one is mainly dependent on the local HF concentration at the pore tip. As a consequence, if we are in the presence of columnar structures, it's because the oxide removal is considerably faster than the electrochemical oxidation. Then, the holes diffusing to the interface are consumed at the pore tips.

### Surface defect revelation on si face

**Figure 7:**
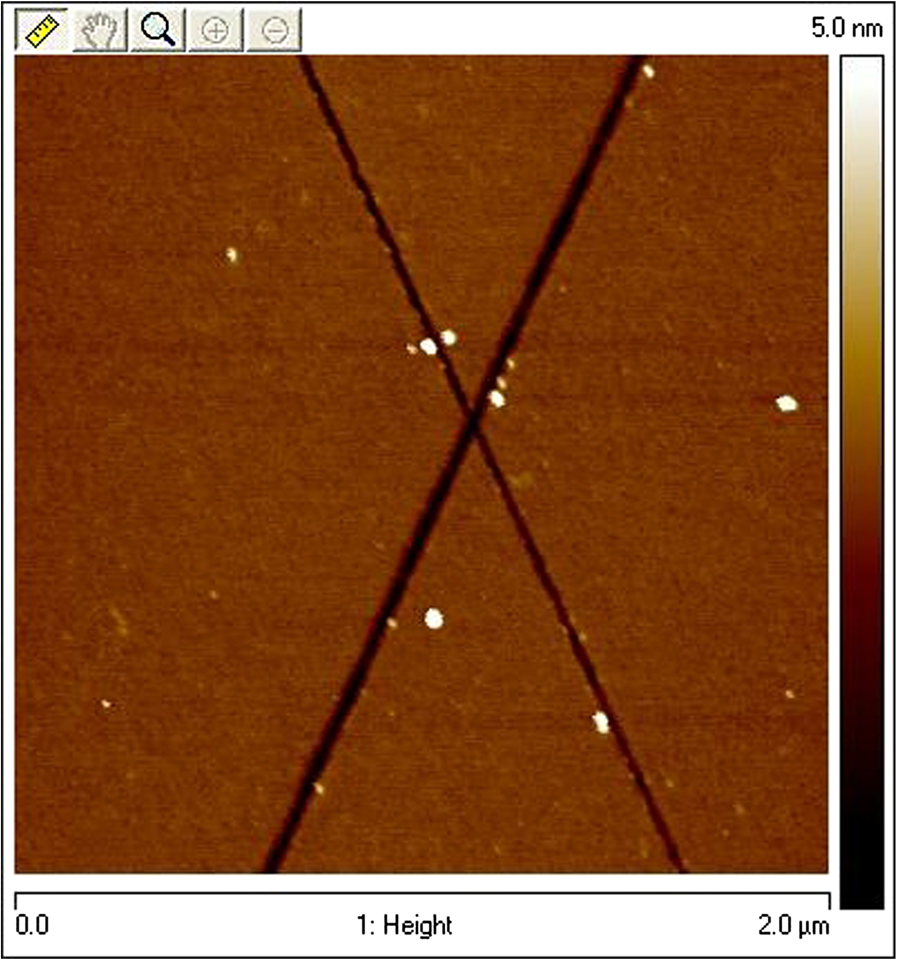
**AFM plan view of CMP scratch marks revealed by electrochemical etching.** The anodization was performed during 45 s at 20 mA.

**Figure 8:**
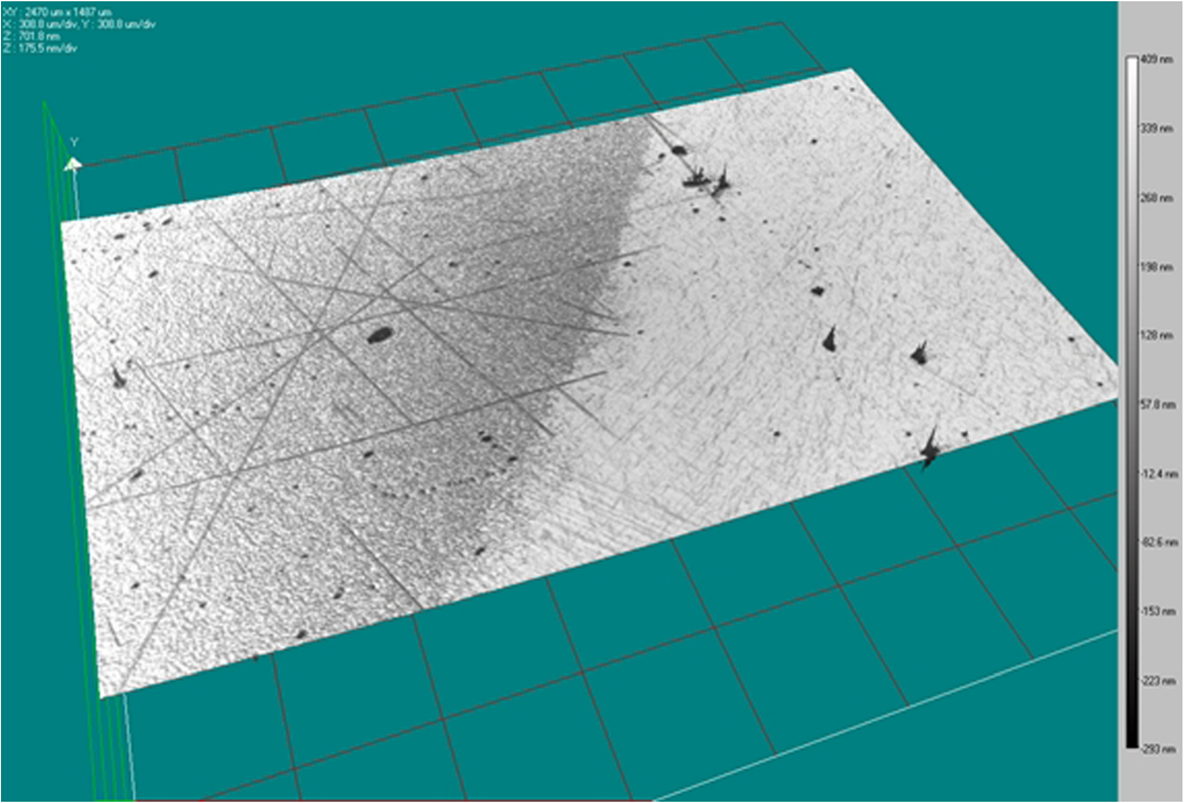
**3D view using optical profilometry of a sample surface etched during 45 s at 20 mA.** The limit between the exposed area where the polishing defects are revealed and the protected one is clearly visible.

It's well known that usually a cap layer is observable at the top of the porous layers [17]. In our case, some nanometric holes are also visible on the surface. They correspond to the opening of some pores which grow through the substrate. The low density observed for these holes and their reduced size are probably at the origin of the very significant diffusion phenomenon which appears into the pores. Moreover, the pores initiate preferentially where the chemical and mechanical polishing (CMP) creates surface scratches (Figure 7). These defects are invisible before the electrochemical reaction or on protected areas (Figure 8) and can be revealed for a short reaction duration, 45 s for our example. If the surface is only mechanically polished, the nucleation is homogeneous on the surface. This observation has been confirmed by atomic force microscopy (AFM) and scanning electron microscopy (SEM). This property can be used for CMP quality inspection on 4 H-SiC wafers.

## Conclusion

In this paper, we've demonstrated the feasibility to achieve homogeneous porous silicon carbide layers from highly doped 4 H-SiC wafers without UV illumination. In particular, we put into relief the electrochemical etching behavior between the Si and C faces. We've shown also the morphologies at different depths and with various current densities. Finally, we've presented some interesting consequences of the surface state on the pore nucleation.

## Competing interests

The authors declare that they have no competing interests.

## Authors' contributions

GG wrote the manuscript and performed the porous SiC. FC, J-F., XS, JB, and MC performed the microscopy (AFM, SEM, optical profilometry) and analysis. All authors read and approved the current manuscript.
